# P-1955. Trends in Skilled Nursing Facility Outbreak Characteristics by Prevalent *SARS-CoV-2* Variant - Los Angeles County, July 2020 - January 2024

**DOI:** 10.1093/ofid/ofae631.2114

**Published:** 2025-01-29

**Authors:** Brendan O’Shea, Amanda Neikirk, Israa Khanqadri, Camellia Babaie, Zachary A Rubin, Prabhu Gounder

**Affiliations:** Los Angeles County Department of Public Health, Los Angeles, California; Los Angeles County Department of Public Health, Los Angeles, California; Los Angeles County Department of Public Health, Los Angeles, California; Los Angeles County Department of Public Health, Los Angeles, California; Los Angeles County Department of Public Health, Los Angeles, California; Los Angeles County DPH, Los Angeles, California

## Abstract

**Background:**

Los Angeles County (LAC) skilled nursing facilities (SNFs) experienced recurrent COVID-19 surges coinciding with the emergence of new SARS-CoV-2 variants. The impact of new SARS-CoV-2 variants on SNF outbreaks relative to other measures, such as the use of COVID-19 vaccines and policies to control COVID-19, in SNFs is unknown. We described trends in LAC SNF COVID-19 outbreak size and duration by SARS-CoV-2 variants detected.

**Figure:**
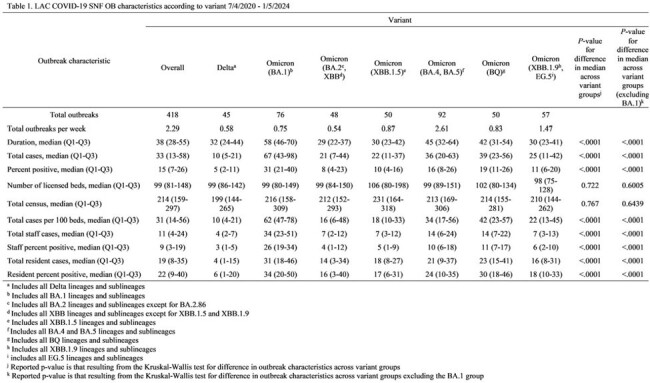

**Methods:**

All SNF COVID-19 outbreaks, defined as > 1 case in a resident, were reportable to LAC Department of Public Health (DPH); SNFs were required to follow DPH recommendations for control including testing all staff and residents weekly and offering COVID-19 vaccines according to national guidelines. An outbreak ended when no cases were detected for > 14 days. We obtained SARS-CoV-2 isolates from respiratory specimen for whole genome sequencing when possible and attributed outbreaks to the predominant SARS-CoV-2 variant detected among sequenced cases. Kruskal-Wallis test was conducted to assess for differences in outbreak characteristics by variant.

**Figure:**
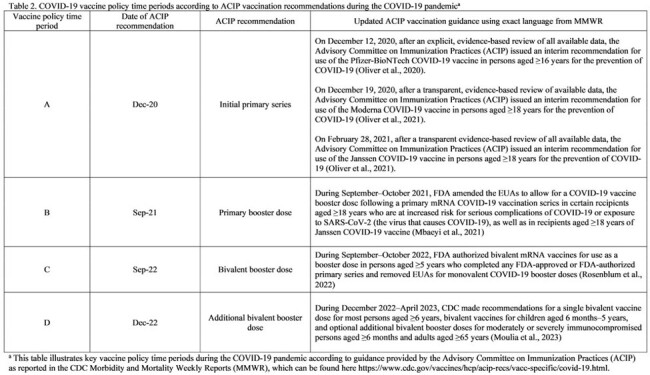

**Results:**

We analyzed characteristics of 418 LAC SNF outbreaks with > 1 sequenced case during 7/4/2020–1/5/2024 (Table 1). Median number of cases/100 licensed beds ranged from 10 (1^st^ quartile [Q1]–3^rd^ quartile [Q3]: 5–21) for Delta- to 62 (Q1–Q3: 47–78) for Omicron (BA.1)-associated outbreaks. Median outbreak duration ranged from 29 days (Q1–Q3: 22–37) for Omicron (BA.2/XBB)- to 58 days (Q1–Q3: 46–70) for Omicron (BA.1)-associated outbreaks. The median licensed bed size of outbreak-associated SNFs was similar across variant groups.

**Figure:**
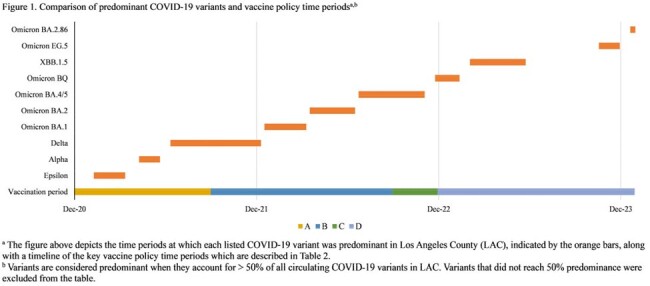

**Conclusion:**

Our results indicated that variations in SNF outbreak size and duration by SARS-CoV-2 lineage did not ecologically correlate with changes in vaccine policy (Table 2 and Figure 1) or with DPH recommendations for controlling COVID-19 outbreaks in SNFs (which remained consistent during the evaluation period). Our results provide additional evidence that certain SARS-CoV-2 variants are more transmissible (Table 3); conducting genomic surveillance can guide public health policymakers and SNF providers on implementing enhanced measures to mitigate COVID-19 outbreaks.

**Figure:**
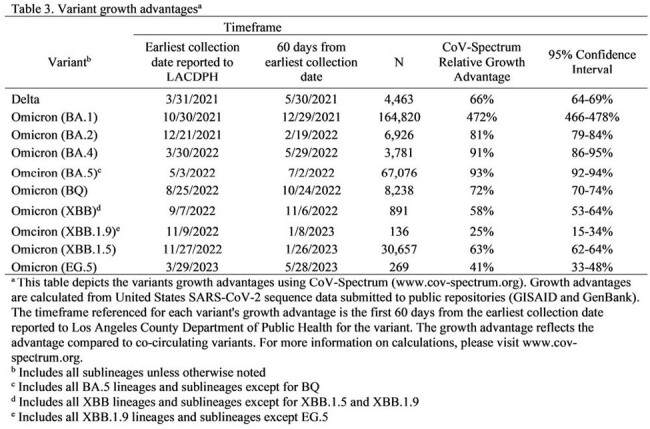

**Disclosures:**

All Authors: No reported disclosures

